# Prognostic Value of Preoperative Serum Levels of Periostin (PN) in Early Breast Cancer (BCa)

**DOI:** 10.3390/ijms160817181

**Published:** 2015-07-28

**Authors:** Pier Vitale Nuzzo, Alessandra Rubagotti, Francesca Argellati, Antonio Di Meglio, Elisa Zanardi, Linda Zinoli, Paola Comite, Michele Mussap, Francesco Boccardo

**Affiliations:** 1 Academic Unit of Medical Oncology, IRCCS San Martino University Hospital—IST National Cancer Research Institute, Largo Rosanna Benzi 10, 16132 Genoa, Italy; E-Mails: piervitale.nuzzo@libero.it (P.V.N.); alessandra.rubagotti@unige.it (A.R.); francesca.argellati@libero.it (F.A.); antonio.dimeglio@rocketmail.com (A.D.M.); elisa.zanardi@unige.it (E.Z.); linda.zinoli@libero.it (L.Z.); 2 Department of Medicine, School of Medicine, University of Genoa, Largo Rosanna Benzi 10, 16132 Genoa, Italy; 3 Department of Laboratory Medicine, IRCCS San Martino University Hospital—IST National Cancer Research Institute Largo Rosanna Benzi 10, 16132 Genoa, Italy; E-Mails: paola.comite@libero.it (P.C.); michele.mussap@hsanmartino.it (M.M.)

**Keywords:** periostin protein, breast neoplasms, extracellular matrix proteins, prognosis, biomarkers, enzyme-linked immunosorbent assay

## Abstract

PN is a secreted cell adhesion protein critical for carcinogenesis. Elevated serum levels of PN have been implicated as playing an important role in different types of cancer, and a few reports suggest a potential role as a prognostic marker. We evaluated the prognostic significance of preoperative serum PN concentration in patients with BCa receiving curative surgery. Enzyme-Linked Immunosorbent Assay (ELISA) was performed to determine the preoperative serum PN level in 182 patients. The correlations between serum PN concentration with clinical pathological features and PN expression in primary tumor samples were analyzed. The prognostic impact of serum PN levels with all-cause and BCa-specific mortality was also investigated. Appropriate statistics were used. Elevated serum PN levels were significantly associated with patient age (*p* = 0.005), adjuvant systemic therapy (*p* = 0.04) and progesterone receptor (PgR) status (*p* = 0.02). No correlation between PN preoperative serum levels and other clinical-pathological parameters, including either the epithelial or the stromal PN expression of primary tumor or the combination of the two, was found. Similarly, no association between serum PN levels and either all-cause or BCa-specific mortality was found. However, subgroup analysis revealed a correlation between higher PN serum levels and all-cause mortality in patients with node-negative disease (*p* = 0.05) and in those with a low PgR expression (*p* = 0.03). Higher levels of serum PN were also found to correlate with BCa-specific mortality in the subgroup of patients who did not receive any adjuvant systemic therapy (*p* = 0.04). Our findings suggest that PN was detectable in the serum of early BCa patients before surgery and increased base-line serum levels predicted worse long-term survival outcomes in specific subgroups of patients.

## 1. Introduction

While the incidence of breast cancer (BCa) is still growing up, since the early 1990s there has been a slow but progressive decrease in BCa-specific mortality [1], probably as the result of both mammographic screening and a multimodal therapeutic approach [2]. Nevertheless, BCa remains the first leading cause of cancer-related death in the female population. Identifying women who might derive the most benefit from multimodal treatments is still an open task. In fact, the use of the prognostic information provided by clinical pathological features or, more recently, by gene signatures is not fully satisfactory for this purpose [3] and the great majority of patients still receive unhelpful treatments and are exposed to undue toxicities. For several decades it has been debated whether and how circulating tumor markers might improve the prognostic information provided by the phenotypic characteristics of the primary tumor. Unfortunately neither carcinoembryonic antigen (CEA) nor carbohydrate antigen 15.3 (CA 15.3), the most commonly used circulating biomarkers to monitor BCa, have been found to be adequately affordable in predicting a patient’s prognosis following local treatment [4,5]. Therefore, researchers’ attention has been focused on several alternative markers; none of them, however, has been proven so far to be effective in this regard [6,7]. Recently increasing attention has been paid to the extracellular matrix (ECM) proteins, which have been demonstrated to play a crucial role in driving carcinogenesis [8]. The crosstalk between cancer cells and ECM proteins, secreted by the neighboring stromal cells, does in fact create a supportive microenvironment that contributes to sustaining tumor growth and spreading [8].

Periostin (PN) is a protein produced and secreted by the fibroblasts as a major component of ECM, where it is involved in the regulation of intercellular adhesion [9]. This protein is thought to play a critical role in carcinogenesis and metastasization by interacting with integrins as well as with other signals mainly via the PI3-K/AKT pathway [10]. Moreover, PN was shown to be a marker and an inducer of epithelial-mesenchimal transition (EMT), a process responsible for the dissemination of primary tumor cells to the sites of metastasis and for the differentiation program leading to the increase in malignant behavior of tumors [11]. It has been reported that PN is frequently overexpressed in various types of human cancer tissues, including breast, prostate, colon, lung, ovarian, head and neck cancer, melanoma, and neuroblastoma [10]. PN is also detectable in biologic fluids, either in healthy people or in cancer patients [12,13,14,15,16]. The role of circulating PN in sustaining tumor progression in cancer patients has been neither fully established nor investigated for the possible clinical implications.

A few studies have evaluated the prognostic significance of PN serum levels in patients affected by solid tumors [12,13,14,15,16] and, at least at our best knowledge, only one study has investigated this aspect in a small group of patients affected by BCa at different disease stages [17]. Lack of adequate information in this regard prompted us to take advantage of a previous study on the prognostic value of the tissue expression of PN in a selected cohort of BCa patients for whom at least one serum aliquot was collected at the time of surgery and cryopreserved [18]. This provided us with the opportunity to assess the prognostic role of serum PN concentration in the cohort patients, focusing on the correlation of preoperative serum PN levels with clinical pathologic variables and tissue expression of PN in primary tumor samples. The association between serum PN levels and patient mortality was also investigated.

## 2. Results

The serum levels of PN in the 182 patients forming the cohort in this study ranged from 43.7 pg/mL to 1034.0 pg/mL, with a median value of 219 pg/mL; this value was arbitrarily selected as the cut-off for statistical comparisons (see statistical analysis section). Patient demography is summarized in Table 1.

**Table 1:** Main characteristics of study patients (N = 182).

Variables	No. of Patients (%)
**Age at surgery, years**	
Median (range)	58 (31–84)
**Menopausal status**	
Pre-menopausal	58 (31.9)
Post-menopausal	124 (68.1)
**Tumor size: cm in diameter**	
≤2	89 (48.9)
>2	93 (51.1)
**Stage**	
I	69 (37.9)
II	76 (41.8)
III	37 (20.3)
**ER status**	
Poor (<10% of stained cells)	40 (22.0)
Rich (≥10% of stained cells)	142 (78.0)
**PgR status**	
Poor (<10% of stained cells)	80 (44.0)
Rich (≥10% of stained cells)	102 (56.0)
**Ki-67**	
Low (<14% stained cells)	85 (46.7)
High (≥14% stained cells)	97 (53.3)
**HER2 status**	
Negative	166 (91.2)
Positive	16 (8.8)
Phenotype *	
Luminal A	60 (33.1)
Luminal B (HER2-neg)	73 (40.1)
Luminal B (HER2-pos)	9 (4.9)
HER2 positive (non-luminal)	7 (3.8)
Triple negative	33 (18.1)
Adjuvant systemic therapy **	
Delivered	104 (57.1)
Undelivered	78 (42.9)
**PN epithelial (E) expression**	
0% immunostained cells	111 (61.0)
≥1% immunostained cells	71 (39.0)
**PN stromal (S) expression**	
<90% immunostained cells	94 (51.6)
≥90% immunostained cells	88 (48.4)
**E/S PN expression**	
E ≥ 1% S < 90%	31 (17.0)
E = 0 S < 90%	63 (34.6)
E = 0 S ≥ 90%	48 (26.4)
E ≥ 1% S ≥ 90%	40 (22.0)

* definition of intrinsic subtypes of breast cancer according to [3]; ** either chemotherapy or endocrine therapy, or both.

### 2.1. Correlation of Serum PN Level with Clinical Pathological Features and Tissue PN Expression

PN serum levels were not correlated with the clinical pathological parameters selected for the present analysis, except for tumor PgR status (*p* = 0.02), patient age at surgery (*p* = 0.005), and adjuvant systemic therapy (*p* = 0.04). No relationship was found between the serum PN levels and either the corresponding epithelial or stromal PN expression or the PN phenotype obtained by combining the expression of PN in both compartments (Table 2).

**Table 2:** Correlation of preoperative serum PN median level with clinical pathological features and tissue PN expression.

Variables	≤219 pg/mL (%)	>219 pg/mL (%)	*p*
**Median age at surgery, years**			
≤58	57 (62.6)	38 (41.8)	
>58	34 (37.4)	53 (58.2)	0.005
**Menopausal status**			
Pre-menopausal	32 (35.2)	26 (28.6)	
Post-menopausal	59 (64.8)	65 (71.4)	0.3
**Tumor size: cm in diameter**			
≤2	45 (49.5)	44 (48.4)	
>2	46 (50.5)	47 (51.6)	0.8
**Stage**			
I	33 (36.3)	36 (39.6)	
II	40 (44.0)	36 (39.6)	
III	18 (19.7)	19 (20.8)	0.8
**Nodal status**			
Node-negative	44 (48.4)	49 (53.8)	
Node-positive	47 (51.6)	42 (46.2)	0.4
**ER status**			
Poor (<10% of stained cells)	24 (26.4)	16 (17.6)	
Rich (≥10% of stained cells)	67 (73.6)	75 (82.4)	0.1
**PgR status**			
Poor (<10% of stained cells)	48 (52.7)	32 (35.2)	
Rich (≥10% of stained cells)	43 (47.3)	59 (64.8)	0.02
**Ki-67**			
Low (<14% stained cells)	42 (46.2)	43 (47.3)	
High (≥14% stained cells)	49 (53.8)	48 (52.7)	0.8
**HER2 status**			
Negative	81 (89.0)	85 (93.4)	
Positive	10 (11.0)	6 (6.6)	0.3
Phenotype *			
Luminal A + Luminal B (HER2-neg)	25 (27.5)	35 (38.5)	
Others **	66 (72.5)	56 (61.5)	0.1
Adjuvant systemic therapy ***			
Undelivered	32 (35.2)	46 (50.5)	0.04
Delivered	59 (64.8)	45 (49.5)	
**PN epithelial (E) expression**			
0% immunostained cells	57 (62.6)	54 (59.3)	
≥1% immunostained cells	34 (37.4)	37 (40.7)	0.6
**PN stromal (S) expression**			
<90% immunostained cells	46 (50.5)	48 (52.7)	
≥90% immunostained cells	45 (49.5)	43 (47.3)	0.7
**E/S PN expression**			
E ≥ 1% S < 90%	15 (16.5)	16 (17.6)	
E = 0 S < 90%	31 (34.1)	32 (35.2)	
E = 0 S ≥ 90%	26 (28.6)	22 (24.1)	
E ≥ 1% S ≥ 90%	19 (20.8)	21 (23.1)	0.9

* definition of intrinsic subtypes of breast cancer according to [3]; ** Luminal B (HER2-pos) + HER2 positive (non-luminal) + triple negative; *** either chemotherapy or endocrine therapy, or both.

### 2.2. Correlation of Serum PN Levels with All-Cause Mortality and BCa-Specific Mortality

**Figure 1:**
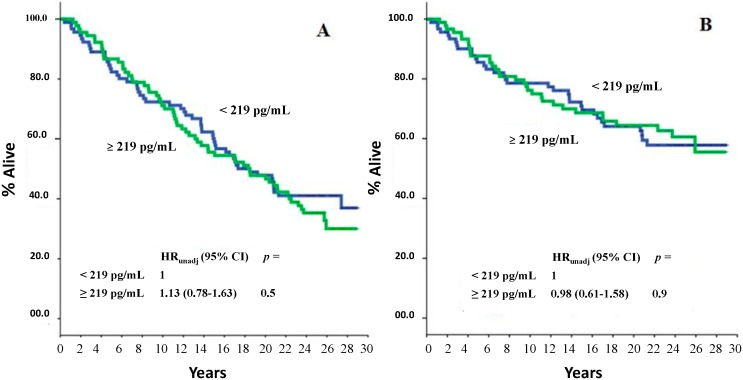
All-cause (**A**) and BCa-specific mortality (**B**) of study patients as a function of serum PN values. The median value, corresponding to 219 pg/mL, was used as an arbitrary cutoff. *HR*: hazard ratio; 95% *CI*: 95% confidence interval.

**Figure 2:**
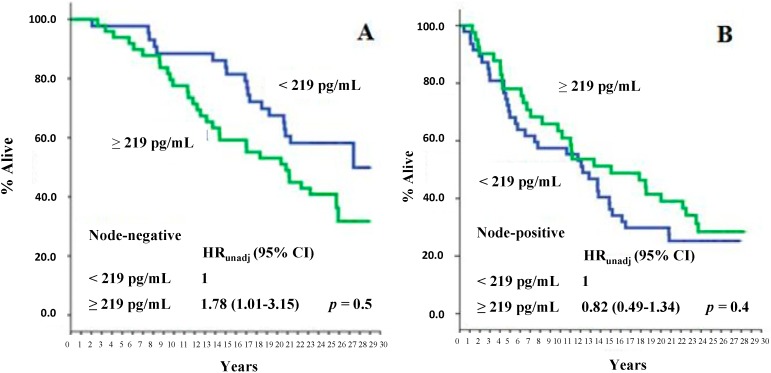

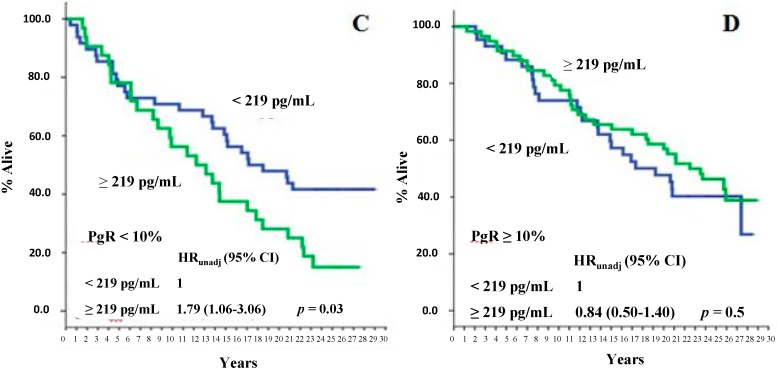
All-cause mortality of study patients as a function of serum PN values, according to nodal status (**A**,**B**) and PgR status (**C**,**D**). The median PN value, corresponding to 219 pg/mL, was used as an arbitrary cutoff. *HR*: hazard ratio; 95% *CI*: 95% confidence interval.

**Figure 3:**
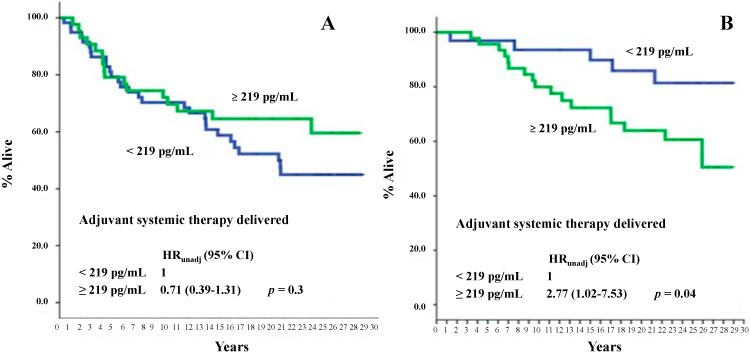
BCa-specific mortality of study patients as a function of serum PN values according to adjuvant systemic treatment (**A**,**B**). The median value, corresponding to 219 pg/mL, was used as an arbitrary cutoff. *HR*: hazard ratio; 95% *CI*: 95% confidence interval.

At a median follow-up time of 225 months (range: 5–353 months), 114 deaths were recorded, of which 67 were BCa-related. As is shown in Figure 1A,B, no association between PN serum levels and either all-cause or BCa-specific mortality was found. However, subgroup analysis showed that there was a statistically significant association between higher serum PN levels (*i.e.*, ≥the median level) and all-cause mortality in patients with node-negative disease (*p* = 0.05) and in those with poor (*i.e.*, <10% stained cells) PgR tumors (*p* = 0.03) (Figure 2A,C). No statistically significant correlation of serum PN levels with any of the other variables was evident relative to BCa-specific mortality, except for a correlation with adjuvant systemic therapy (*p* = 0.04 in favor of the patients who did not receive adjuvant systemic treatments, Figure 3B).

## 3. Discussion

### 3.1. General Remarks

Our study has some intrinsic limitations, and for this reason our data should be managed carefully.

Major limitations are represented by the relatively small size of the cohort and by the retrospective nature of the study. Both conditions can affect the statistical power of the study. Nevertheless, this is by far the largest cohort of patients among those studies that evaluated the serum concentration of PN in cancer patients. The median value of serum levels of PN, measured by the ELISA technique, corresponded to 219 pg/mL, a value highly comparable with PN median values found by Hong *et al.* [16] in non-small cell lung cancer (24,284 pg/mL). However, various discrepancies related to the serum concentration of PN detectable in cancer patients exist among previous available studies [12,13,14,15,16,17]. These differences can be explained not only by the different tumor type or tumor stage affecting the patients included in the different studies [12,13,14,15,16], but also by the poor comparability of results due to the lack of commercial assays standardization. In fact, a number of critical issues may affect results: the lack of an international standard calibrator for PN, the heterogeneity of antibodies among commercial kits, including the affinity and the avidity, differences in analytical methods (ELISA *vs.* chemiluminescence), and, finally, variability due to manual procedures in such analytical protocols (e.g., ELISA).

### 3.2. Correlation of Serum PN Level with Clinical Pathological Features and Tissue PN Expression

In our study serum PN levels showed no relationship with established clinical pathological parameters (except for a weak correlation with tumor PgR status) as well as with PN tissue expression, while a significant correlation with patient age and adjuvant systemic therapy was found.

The significant relationship between serum levels of PN and age in our cohort could derive from multiple causes. Elevation in serum levels of PN could be attributable to normal or abnormal bone turnover or to other factors (including myocardial, vascular, and skeletal muscle injuries) [10,11], unrelated to the presence of the neoplasm. Unfortunately, information about these factors and other putative confounding factors such as smoking, infections, or other aging-related diseases is missing and cannot be obtained after such a long follow-up time (median follow-up time: 225 months) and the majority of the cohort patients died (114 out of 182).

Available literature data show that serum PN levels do not correlate with clinical-pathologic parameters in lung cancer, colorectal cancer, thymoma, and hepatocellular carcinoma [12,14,15,16]. However, a significant difference in serum PN levels was shown in patients with advanced stage III/IV colorectal cancer, stage IV thymoma, and metastatic hepatocellular carcinoma, as compared to earlier stages [12,14,15]. Comparably, Sasaki *et al.* [17] reported on the presence of elevated levels of serum PN in BCa patients affected by bone metastases, suggesting that serum PN might represent a marker of bone metastases in these patients [17]. We were not able to evaluate differences in terms of serum PN levels between early BCa patients and stage IV patients, as our samples were obtained pre-operatively only from non-metastatic BCa patients. The presence of elevated levels of serum PN in metastatic cancer patients could support the hypothesis that the greatest amount of circulating PN might be released into the blood stream from the metastatic sites rather than from the primary tumor. This assumption appears to be supported by the lack of any relationship between PN primary tumor levels and serum PN levels in our study and by the results of a previous experimental study on a BCa mouse model showing that the PN stromal response to the attachment of cancer stem cells at the metastatic niche level might contribute to the circulating levels of PN in the blood [19].

### 3.3. Correlation of Serum PN Level with All-Cause Mortality and BCa-Specific Mortality

No association between PN serum levels and either all-cause or BCa-specific mortality was found in our study.

However, subgroup analysis showed that there was a statistically significant association between higher serum PN levels and all-cause mortality in specific subgroups. For instance, we found that higher serum levels of PN were significantly associated with a higher all-cause mortality risk in node-negative but not in node-positive patients. This was not expected. However, the putative confounding role of adjuvant systemic therapies should be taken into account in interpreting these findings. In fact, at the time cohort patients were operated on, only those showing nodal involvement were usually treated prophylactically with either endocrine therapy or chemotherapy, or both. The effect of systemic therapies on micro-metastatic residual disease might well blunt the prognostic value of PN serum levels in this patient subgroup. This putative bias is lacking in node-negative patients, who were not candidates for either form of adjuvant systemic treatment. This assumption appears to be supported by the fact that a positive association of higher PN serum levels with a worse outcome in terms of BCa-specific survival was also found in our study. The association found between serum PN levels and tumor PgR expression (higher serum PN levels were associated with a poorer survival outcome in patients with PgR-poor tumors but not in those with PgR-rich tumors) could again be explained by the putative confounding effect of systemic therapy, since adjuvant endocrine therapies were commonly given to patients with PgR-positive tumors.

## 4. Experimental Section

### 4.1. Patient Selection and Sample Collection

Tissue PN expression had been previously evaluated in the archival material relative to 200 patients diagnosed with BCa and operated at our Institute between 1985 and 1990. Preliminary results were presented at the ESMO Impakt 2015 conference [18]. We found that of these 200 patients for whom it had been possible to update follow-up data, particularly in respect to mortality, serum samples had been obtained from 182 of them just before surgery and stored at −80 °C.

All patients consented to provide blood and to the cryopreservation of relative serum aliquots, and all of them were informed about the experimental nature of the studies that would be performed in the future and had been made aware of the fact that no beneficial effect would be obtained by their participation to the program. The use of archival and cryo-preserved material for the purpose of the present study was approved by the Ethics Committee of Regione Liguria–Section 1. Patient data were managed according to the Italian Data Protection Authority prescriptions [http://www.garanteprivacy.it].

### 4.2. Enzyme-Linked Immunosorbent Assay (ELISA) Evaluations

Patient serum PN levels were measured by ELISA technique using the commercial kit “Periostin Matched Pair Detection Set” (Adipogen International, San Diego, CA, USA), consisting of a 96-well microplate (Nunc MaxiSorp Plate, ThermoFisher Scientific, Waltham, MA, USA). The test was optimized and automated on the multiparameter analyzer (TRITURUS^®^, Pantec, Torino, Italy). Briefly, microplates were coated with the specific antibody anti-PN (2 μg/mL, 100 µL), diluted in phosphate-buffered saline (PBS), and incubated overnight at 4 °C. The remaining protein-binding sites were blocked by keeping them in a buffer (2% bovine serum albumin in PBS) for 2 h at 25 °C, and then washed five times with 300 µL/well PBS containing 0.1% Tween-20. Subsequently, standard dilutions and serum samples were added and incubated for 2 h at room temperature.

After washing, plates were then incubated for 1h at room temperature with detection antibody (0.1 μg/mL, 100 µL/well) and, after washing, the detection antibody was labeled with horseradish peroxidase-labeled streptavidin (100 µL/well) for 30 min at room temperature. The enzyme reaction was activated by adding reaction solution (3,3',5,5'-tetramethylbenzidine) for 20 min at room temperature. The chromogenic reaction was stopped by the addition of 2 M H_2_SO_4_ and the absorbance was measured at 450 nm (primary wavelength) and 550 nm (secondary wavelength). The absorbance at 550 nm was subtracted from the absorbance at 450 nm. PN concentrations were calculated from the standard curve generated by a curve-fitting program (4PL). PN concentrations were expressed as pg/mL and the detection limit was 78 pg/mL. All samples were assayed in duplicate.

### 4.3. Statistical Analysis

Median serum PN values have been arbitrarily chosen for statistical analysis. In fact the discriminant power of tertile or quartile values was not better nor did ROC analysis allow us to discriminate values more strictly associated with individual mortality risk.

The chi-square test was used to study the relationship between median preoperative PN level in the serum and the following clinical-pathological parameters: patient age at surgery (≤58 *vs.* >58 years, *i.e.*, median age), menopausal status (pre- *vs.* post-), T size (≤2 cm *vs.* >2 cm), nodal status (negative *vs.* positive), tumor stage (I *vs.* II *vs.* III), estrogen-receptor (ER) status (<10% *vs.* ≥10%), PgR status (<10% *vs.* ≥10%), Ki-67 proliferation index value (<14% *vs.* ≥14%), HER2 status (negative *vs.* positive), tumor phenotype (luminal A + luminal B HER2-neg *vs.* the other immune-phenotypes), and adjuvant systemic therapy (delivered *vs.* undelivered).

The chi-square test was also used to correlate preoperative PN serum levels with either the epithelial or the stromal PN tissue expression, or with any of the four phenotypes arbitrarily obtained on the basis of both epithelial and stromal expression and showing different mortality probabilities as described in the previously mentioned study [18]. Concerning the correlation with all-cause and BCa-specific mortality, curves were constructed through the cumulative incidence function estimated by the Kaplan–Meier method and compared by way of the log-rank test [20]. All *p* values were two-tailed. The IBM software Statistical Package for Social Sciences (SPSS) version 21.0 forWindows (SPSS Inc., Chicago, IL, USA) and STATASE11 were used for data analysis.

## 5. Conclusions

In spite of the limitations already mentioned in the general remarks, our findings show that PN is present at detectable levels in the serum of early BCa patients. The production of circulating PN does not seem to occur at primary tumor sites or to be in any way correlated with PN tissue expression. Though PN serum levels do not appear to correlate with mortality in the whole cohort, higher serum levels of PN predicted worse survival outcomes in specific subgroups of patients, namely in those who, by virtue of their nodal and PgR status, did not receive any form of adjuvant systemic treatment. Further investigations on the biological role of serum PN in early BCa patients and on its putative clinical significance are warranted.
